# Inclusive Environmental Decision-making in a Developing Nation: Insights from the Ogoni Remediation Project, Niger Delta, Nigeria

**DOI:** 10.1007/s00267-023-01885-y

**Published:** 2023-09-29

**Authors:** Kabari Sam, Tubodenyefa Zibima

**Affiliations:** 1 School of Environment, Geography and Geoscience, University of Portsmouth, Burnaby Road, Portsmouth, PO1 3QL UK; 2 Department of Political Science, Niger Delta University, Wilberforce Island, Bayelsa, Nigeria

**Keywords:** Communication, Cultural beliefs, Gender policy, Inclusion, Niger Delta

## Abstract

Greater involvement of women is globally believed to enhance environmental management outcomes. Despite being disproportionately affected by environmental degradation primarily caused by oil spills in the Niger Delta region of Nigeria, women in the region are often excluded in environmental decision-making processes. Women involved in farming and fishing face increased vulnerability to contaminated land, food insecurity and conflicts driven by environmental degradation. Using a mixed approach, women, lawmakers, policymakers, regulators, civil society organizations, environmental management practitioners, and government agents responsible for environmental remediation were engaged through interviews, focus group discussions and questionnaires to examine women’s inclusion in environmental decision-making and governance in the Ogoni contaminated land remediation project in the Niger Delta region of Nigeria. The results indicate that lack of trust and confidence in drivers of the environmental decision-making process could affect women’s participation and involvement in environmental management. Although cultural beliefs and practices impede the participation of women in the region, their actual participation does not independently translate into inclusion in environmental decision-making due to limited capacity, confidence and trust in the process. Creating platforms for capacity building, developing gender policy, adapting appropriate communication strategies, initiating women networks and strengthening social cohesion could enhance women involvement in environmental decision-making in regions with similar cultural context to the Niger Delta region.

## Introduction

Globally, the involvement of women in the environment sector is widely recognized especially in farming, fishing, biodiversity conservation and designing and implementing solutions for environmental management. However, women’s involvement and representation in decision-making processes that relate to their livelihoods, families and environmental-wellbeing are often relegated (Grillos 2018; IUCN 2015; FAO 2014). Traditional belief systems that delineate gender roles reflects men’s involvement and dominance in commercial activities, and Women’s involvement and dominance in domestic activities has restricted their ability to be considered and fully engaged in the environmental decision-making process (IUCN 2015; FAO 2013; Witinok-Huber et al. 2021). In addition, due to men’s involvement in the commercial use of natural resources, which contributes to formal economy, men become visible to policy makers and other stakeholders than women. As a result of this dominance, less attention is given to the dimensions of skills and knowledge systems contributed by women in the management and sustenance of the environment (FAO 2011; 2014).

Women engage and interact with the environment daily in their domestic and economic roles. In developing countries such as Nigeria, women are typically responsible for subsistence agriculture including food harvesting and production, growing crops, shellfish harvesting (Ugwu 2019; Amusan et al. 2021). As they cultivate the soil and observe changes in soil visual characteristics and agricultural yields during harvest, they understand threats of a compromised soil to their yields, and potential impacts on the health of those that consume agricultural produce cultivated on harmful soil. Thus, with their indigenous knowledge of soil texture and color, they could contribute to the characterization of the risks and impacts of environmental degradation to the environment, when given the opportunity. Grillos (2018) provided evidence of sustainable environmental management as a result of robust women’s participation in environmental decision-making and suggested the need for creating a participatory and conducive environment for stakeholder interaction.

However, despite womens’ interaction with, and dependence on, the environment, (e.g., harvesting shellfishes on sediments) (Chris et al. 2023), their involvement and participation in environmental decision-making is informal and mostly insignificant (Zabbey 2009; Uduji and Okojo-Obasi 2020; Colulter et al., 2019). Evidence of gender inclusion in the global south is mostly at the participation level with no significant influence on the outcome of environmental management decisions. For example, in the evaluation of interventions to increase drought management preparedness in northern Kenya, Grillos (2018) reported an increase in women’s awareness and participation in structured decision-making process, however, this did not translate to meaningful influence on the final outcomes of the decision. Similarly, Devkota (2020) investigated opportunities for using the principles of social inclusion to enhance deliberations and the participation disadvantaged groups (especially women) in community forestry program in Nepal. The research indicated that despite an increase in participation, women are yet to influence the outcomes of final decisions. Also, amidst many environmental issues confronting women in the Republic of Liberia, many women are not involved in the decision-making process that relates to them (IUCN 2015; Coulter et al. 2019; Witinok-Huber et al. 2021). Although social exclusion is a global challenge, it is heightened in regions where traditional beliefs and culture restrict the participation of women in decision-making processes (Witinok-Huber et al. 2021). The under-acknowledgement of women’s roles could limit the potential economic and societal benefits their contributions bring to environmental management decisions (IUCN 2015). In this research, women participation in environmental remediation and restoration of the Ogoniland, in the Nigerian Niger Delta, is investigated to provide insights for influencing and improving environmental decisions and management.

### Environmental Degradation in Nigeria and the Role of HYPREP

Environmental degradation is a growing problem in developing countries particularly those dependent on natural resource mining (Sam 2016). Reasons for this include that extant legislations are piecemeal and often too weak to effectively ensure adequate environmental protection (Olawuyi and Zibima 2018; Sam et al. 2023), resulting in the local populations suffering from the impacts of environmental degradation (UNEP 2011).

In the early 1970s, oil discovery changed the focus and the dependency of Nigeria from agriculture to crude oil. Today, the Niger Delta region of Nigeria is the hub of oil production -the mainstay of the Nigerian economy. Currently, the region contributes over 90% of foreign exchange and about 9% to Nigeria’s Gross Domestic Product (GDP), while agriculture contributes 26.9% to GDP (Statista 2020). Nigeria is Africa’s largest producer of petroleum and the sixth largest producer in the world. Crude oil production capacity of the country, which is concentrated in the Niger Delta (Veraart et al. 2020) and adjoining offshore wells stands at 2.3 million barrels per day. There is at least 38.2 billion barrels of crude oil reserves in Nigeria. Thus, Nigeria’s reliance on crude oil to sustain the national economy will linger, while the consequential impact of oil exploration and exploitation on both the environment and human health in the Niger Delta, will likely continue (Sam and Zabbey 2018).

The environmental impacts of oil operations in the Niger Delta are reportedly caused by anthropogenic activities (e.g., oil theft and bunkering, and artisanal refining) (Kamal and Kutay 2021; Bodo et al. 2020), and equipment failure (e.g., pipeline rupture and engineering failure) (Akinwumiju et al. 2020; Azuazu et al. 2023). Consequently, environmental degradation in the region has led to change in land use, as profitability from several land-based ventures have declined (Sam and Zabbey 2018). Given the change in land use, agricultural activities (i.e., farming and fishing) has been on the decline thereby constraining economic development in the region (Ansah et al. 2022; Eriegha and Sam 2020). This has worsened in the last three decades as crude oil exploration and exploitation in the region is undertaken with considerable disregard for the basic principles of sustainable environmental management (e.g., the precautionary and polluter pays principles) (Akinpelumi et al. 2023), leaving behind potentially polluted sites with various toxic hazardous chemicals (Sam and Ukotije-Ikwut 2020), which has resulted to loss of biodiversity, livelihoods and deprivation, and the general lack of development in host-communities in the region (Ansah et al. 2022; Sam et al. 2017). Lead poisoning as a result of artisanal gold mining in Zamfara state, Nigeria and the attendant public health effects and fatalities provides an evidence of the lethal impacts of natural resource mining on the environment (Bartrem et al. 2022).

In the Ogoni area, massive oil spills resulted in agitations from local communities and the civil society organizations (CSOs) (UNEP 2011). In mid 1990s, the agitations heightened and crystalized in global attention to the region. In late 2008, the Nigerian Government commissioned the United Nations Environment Programme (UNEP) to undertake an Environmental Assessment of Ogoniland. In 2011, UNEP submitted a comprehensive report establishing massive environmental and socio-economic impacts of the oil industry operations in Ogoniland. In 2012, the Nigerian Government responded to recommendations in the UNEP Report by establishing the Hydrocarbon Pollution Remediation Project (HYPREP), with a mandate to implement recommendations of UNEP on Ogoniland. HYPREP was re-invigorated by the setting up of a Trust fund, and a governance structure required to undertake the clean-up of Ogoniland, as recommended by civil society organizations and the local communities (Sam et al. 2017; Sam et al. 2022).

Over the last five years of HYPREP’s operations in Ogoniland, national and international stakeholders including CSOs, community leaders, media, government and development agencies have expressed concerns on the effectiveness, technical competence and inclusive mechanisms of HYPREP (Sam et al. 2022; Zeeuw et al. 2018). Specifically, the role of women, and their participation is generating a national discourse in the media and local communities. It has been observed in several forums that the activities of HYPREP negates social inclusion (Zeeuw et al. 2018; Sam et al. 2022), resulting in exclusion of women from the decision-making process relating to remediation activities, and makes HYPREP unresponsive to community concerns and expectations.

Considering the nature of HYPREP’s mandates, decision-making processes that encourage exclusion of any segment of the community could have negative implications on community buy-in and ownership for successful remediation and restoration exercise. The potential negative outcomes arising from exclusion, especially women’s exclusion, may impact community support for the project particularly when we consider, for example, women groups that are actively involved in sensitization and awareness creation programs. Women also play critical roles in peace building procedures, especially as it relates to the conduct of youths in the remediation process (Akpan et al. 2014). Most important, women are mostly farmers and dependent on the impacted natural capital for agricultural livelihood. For example, women use proceeds from their agricultural businesses to pay tuition fees for their children, provide daily needs and family support (Pegg and Zabbey 2013; Sam and Zabbey 2018). Thus, women are disproportionately affected with respect to oil spills in impacted communities (Sam et al. 2017b). Therefore, women are critical stakeholders in polluted land remediation decision-making processes for a peaceful exercise, and the restoration of livelihoods and well-being in oil impacted communities.

Given the role of women in environmental decision-making, and the need for diversity of stakeholders (de Siqueira et al. 2021), that will interrogate current practices with regard to the clean-up exercise, this study looks at the state of inclusivity in the environmental remediation process. Specifically, we seek to address the degree of participation and the issues limiting women’s participation in environmental decision-making. Our study seeks to interrogate current levels of women’s participation and influence in decision outcomes, the determination of what outcomes should be and how to go about creating desired outcomes, and the actual roles in the environmental decision-making process in the context of environmental remediation in Nigeria. We intend to provide insights on the peculiarities of the Nigerian context and shared responsibilities towards sustainable environmental management in Nigeria. In the next section, we present a scenario of environmental decision-making in the Niger Delta, followed by the methods and mechanisms adopted to collect data, then the discussion and conclusion of the research.

## Embedding Inclusive Environmental Decision-making in Participatory Environmental Governance

Efforts at achieving sustainable development and environmental protection has recognized public participation in decision making as crucial. The United Nations Agenda 21 acknowledged the necessity of the emergence of new forms of participation if governments intend to actualize environmental protection and social development. The core of the call for newer forms of participation in the decision-making process is to ensure inclusive environmental decision-making that allows different segments of society to participate, influence and determine how environmental outcomes impact their lives (Jänicke and Jörgens 2020).

Encapsulating inclusive environmental decision-making is the idea of participatory environmental governance. The concept and idea of participatory environmental governance has been identified as critical to managing contemporary and emergent environmental issues. Researchers have moved focus to processes of democratizing environmental decision-making and governance (Koontz 2016; Wesselink et al. 2011). Whereas theoretical positions attempt to link improved environmental outcomes to participatory processes and approaches, arguments remain as to the capacity of participatory models leading to sustainable environmental outcomes (Gerlak et al. 2013; Young et al. 2013).

Concerns have been raised around environmental outcomes and participatory processes (Bodin 2017). Conversely, empirical studies have correlated participatory decision-making processes to sustainable environmental outcomes (Biddle and Koontz 2014; Biddle 2017; Newig and Fritsch 2009; Scott 2015). However, critical elements of participatory and inclusive decision-making are the nature of participation, degree of inclusivity, the context of participation and how these shape environmental outcomes on the one hand, and what parameters can be used to mark environmental outcomes determined by participatory input to decision-making.

Participation and inclusive decision-making can be determined through power delegation between the public and the participants, the nature of communication, information exchange and inclusivity; measured by the extent of public involvement in environmental governance (Newig and Rose 2020; Arnstein 2007; Fung 2006). Thus, inclusivity in environmental decision-making, with specific reference to women participation in the Ogoni environmental remediation project, can be measured and framed by three variables. The combination of how much participatory powers are delegated to women groups as a viable segment of the Ogoni community; communication and information flow between traditional governance institutions coordinating the remediation project (HYPREP); and the extent to which women groups are empowered and involved in the environmental remediation process.

## Methods

### The Study Area - The Niger Delta and Ogoniland

**Fig. 1:**
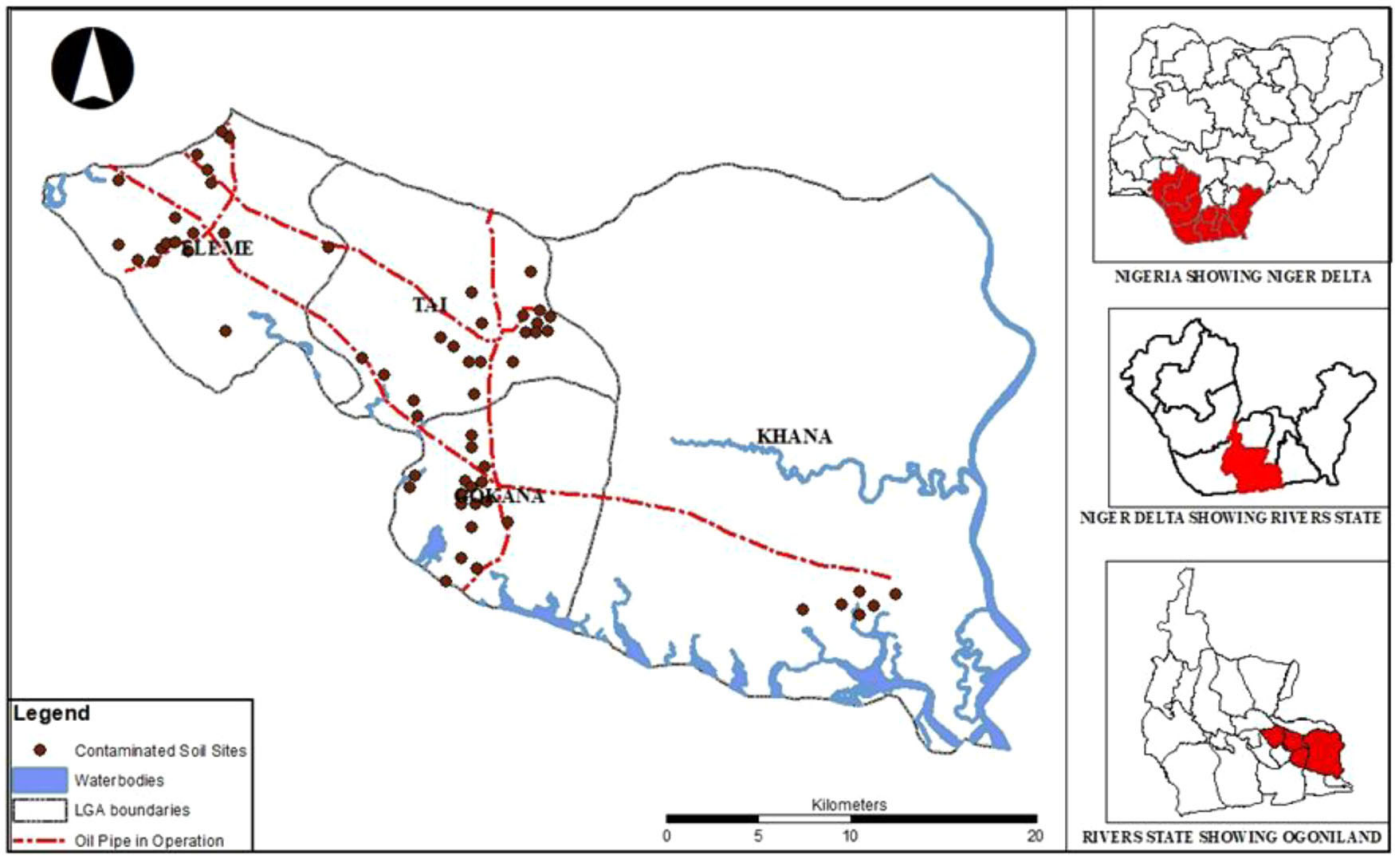
Polluted areas and impacted local government councils in Ogoniland, Rivers state (Adapted from Sam et al. 2022)

The study was conducted in Ogoni, which comprises four local councils in Rivers state, one of the nine states that make up the Niger Delta Region of Nigeria. The Ogoni people popularly called the Ogonis have a population of about 830,000 people in a land mass of 1000 Km^2^ Fig. 1 below has further details. The Ogonis are predominately fishers and farmers (Sam and Zabbey 2018). There are four Local Government Areas (LGA) in Ogoni land; Eleme, Gokan, Khana, Tai. Ogoniland sits on large deposits of crude oil and natural gas; crude oil was discovered in the area in 1958 (Kpae 2021). Ogoni land has been adversely affected by oil spills caused by long-term oil exploration and production activities (Sam et al. 2017), which have impacted farmlands and rivers resulting in biodiversity and livelihood losses with associated unemployment and hardship due to the destruction of the natural resource base in the area (Gimah and Bodo 2019; Sam and Ukotije-Ikwut 2020; Ocholi 2022).

The vegetation over much of the riverine floodplains is made up of arable farmlands, tree crop plantations and patches of naïve species. The arable crops include cassava, yams, maize, pineapple, pepper, rice and leafy vegetables (Bodo and Gimah 2019; Ganabel et al. 2021). The tree and fruit crops include the oil palm, rubber, cocoa, plantain, pawpaw, mango, guava and citrus (Aisien et al. 2021). Thus, the traditional livelihood of the local population in the region is farming and fishing. The basis for agriculture is soil. All crops for human food and animal feed depend on a fertile soil. However, there is an unquantifiable loss of this natural resource by increasing soil pollution occasioned by oil spills in the Niger Delta (Aa et al. 2022). Soil pollution causes significant loses of income, impacts on food security and pose harm to human health (Sam and Zabbey 2018). Women are disproportionately affected given that they are the primary producers and income providers through subsistence agriculture (Naggea et al. 2021). In most coastal communities, women are also involved in fishing activities, even as the men, generally sought and prefer white collar jobs. Traditionally, men are provided the necessary support to acquire basic education which qualifies them for white collar jobs while women are provided informal training including the art of fishing to feign for their household.

Like most communities in the Niger Delta, the Ogonis have lived with chronic oil pollution for over five decades. Studies have shown that government regulation to address oil spills, and technical and logistic capacity to remediate identified sites is limited and the control, maintenance and decommissioning of oilfield infrastructure by oil companies is inadequate (Rim-rukeh 2015; Ite et al. 2016; Olawuyi and Zibima 2018; Ambituuni et al. 2014; Sam et al. 2015; Bartrem et al. 2014). As a result, the Ogoni communities continue to be exposed to extremely high levels of air, soil and (drinking) water pollution that far exceed national and international safety levels (NCF 2006). These pollutions are recurring as a result of poor maintenance, corrosion, faulty equipment, failed clean-up attempts, ‘bunkering’ (i.e., large-scale illegal tapping of oil from pipelines) and artisanal refining (i.e., small-scale, illegal refining of oil) (Naanen 2019), oil pollution is widespread and has become an epidemic in the Niger Delta (NCF 2006).

The study adopted a mixed methods design. Both qualitative and quantitative methods were employed to collect data from target groups. The advantage of the mixed approach is that complementary strategies could be adopted to fit different groups of stakeholders, and thus allow for data triangulation. It also allowed for the collection of embedded data that is at once amenable to statistical analysis and representing lived experiences of respondents.

Based on the research design, respondents for the study were identified from previous studies (e.g., the UNEP Report), and networks (e.g., civil societies network) (Prpich et al. 2019; Sam et al. 2017c). Next, stakeholder mapping (i.e., identification and mapping of stakeholder groups) and community engagements on the remediation project were conducted. This process enabled the identification of critical and key informants for the qualitative data collection phase. The selection criteria were primarily based on experience in the remediation process, understanding of the role of women in decision-making in the context of the remediation exercise and environmental decision-making. Given the nature of the study and the issues explored, respondents included Ogoni women, HYPREP staff, members of CSOs, community leaders, and development practitioners.

### Data Collection and Analysis

Broadly, qualitative data were derived from focused group discussions (FGDs), questionnaires and interviews. Prior to each section, consent was sought and participants were clarified of their rights to leave the process at will. Six FGDs were held with target women population from the Ogoni extraction. Each FGD had 9–12 women in attendance. FGD outcomes were transcribed. Six key informant interviews were conducted with experts and development practitioners who are experienced in the remediation process and gender sensitivity issues. Quantitative data was collected through a guided questionnaire survey using the COMMCARE App (version 8.0) on a cell phone. COMMCARE enabled data protection and confidentiality of participants. The survey questionnaires were distributed in eight (8) selected Ogoni communities cutting across the four LGAs in Ogoniland (Table 1). Communities were selected based on two factors including ongoing remediation activity, and a history of oil contamination. The inclusion criteria for selecting the eight study communities were level of spread and number of oil spill cases and sites, number of HYPREP clean-up sites, and involvement in historical fishing and farming practices. Applying the outlined criteria, two communities meeting these criteria were selected from each of the four Ogoni LGAs (Table 1). The COMMCARE App was used to engage respondents including community women, female youths, farmers, fisherfolks, traders, and female artisans. The app was used to self-administer the questionnaires in the format of a Computer-Assisted Personal Interview (CAPI) (Rao et al. 2020). This allowed for the research assistants to capture responses from respondents directly as a way of overcoming limitations in filling out paper questionnaires.

**Table 1:** LGAs and Selected Communities Sampled in Ogoniland

	LGA	Communities
1	Khana	Kwawa, Kpean
2	Gokana	K-Dere, Mogho
3	Tai	Korokoro, Gio
4	Eleme	Alode, Nsisioken

The total population for the four (4) Ogoni LGAs is given as Eleme LGA: 190,884; Tai LGA: 117,797; Khana LGA: 294,217; Gokana LGA: 228,828. This made for a total Ogoni population of 831,726 (UNEP 2011). The study applied the Yamane formula for determining sample size (Chaokromthong and Sintao 2021; Louangrath 2017) for a finite population using the population value of 831,726. This enabled the researchers to arrive at a sample size of 399.9, approximated to 400 respondents. To ensure representativeness of responses collected across the study area (Ramsy and Hewitt 2005), the research aimed to achieve 50 response from each of the eight communities selected using the inclusion criteria. Community members were identified using a snow ball sampling approach, whereby an initial group of participants shared information about the survey with friends and other community members (Noy 2008; Rizzo et al. 2015; Sam 2016; Sam et al. 2017c). In addition, data saturation was achieved early in the data collection process as additional respondents did not provide new information to the data (Guest et al. 2020; Mwita 2022). In all, 400 respondents were sampled, fifty (50) for each of the study communities using the COMMCARE App between August 21 and October 5, 2020. Out of the 400 sampled respondents in the eight communities, 14 entries were invalid and data from 386 entries were collated for the analysis. With the use of the CAPI approach, respondents who declined participation were not captured, limiting the possibilities of unreturned questionnaires that would be the case with paper questionnaires. The secondary data was collected through the review of gray and peer reviewed literature. At the end of the data collection process, quantitative data collated were analyzed on Microsoft Excel using simple descriptive analysis and presented in Tables and Bar charts.

## Results

### Current Level of Women Participation in Decision-making in the Cleanup Process

As a baseline for the study, the first step sought to evaluate and establish the current level of women participation in decision-making in the overall remediation process in Ogoniland. As an entry point into estimating the level of women participation and influence in the process, the research engaged with women respondents on several aspects of possible participation ranging from actual work on site to community engagement and governance structures for inclusive and collective decision-making such as the Central Representative and Advisory Council (CRAC). As indicated in Table 2, majority of the respondents (98%) stated that women are neither engaged as contractors nor involved in the supply of materials on site (98%), as as they are not part of the monitoring teams (95%).

**Table 2:** Outcomes on the Current Level of Women Participation in the Cleanup Process in Ogoniland

Options	Satisfaction with current level of women participation		Women engaged as contractors		Women employed at sites being cleaned		Women as part of CRAC		Women involved as monitors		Women involved as suppliers	
	Freq	%	Freq	%	Freq	%	Freq	%	Freq	%	Freq	%
Yes	5	1.3	5	1.3	54	14.0	19	4.9	28	7.3	7	1.8
No	381	98.7	381	98.7	332	86.0	367	95.1	358	92.7	379	98.2
Total	386	100	386	100	386	100	386	100	386	100	386	100

Different aspects of women participation were evaluated to ensure a robust determination of the nature and degree of women involvement in the entire remediation process (Table 2). Over 98% of respondents are not satisfied with the current level of women participation in the clean-up process. 98.7% noted that women were not engaged as contractors, or employed at sites being cleaned (86.0%). Over 95% of the sampled population perceived women are not involved in governance and decision-making arms of the project such as CRAC—a conflict resolution committee (Table 2). r involved as site monitors (92.7%). Of the total respondents, 98.2% also noted that women were not engaged as suppliers (Table 2). These results were corroborated by key informant interviews as a respondent (Ogoni Female 1 [OF1]) noted that:

“we were not aware of the cleanup process not until civil society organization visited Eleme in 2017 for awareness and sensitization campaign that the issue of cleanup was for the first time brought to the knowledge of women in Alode Eleme. It was also reported then that a cleaner and a site nurse are the only females from the community working at the site”.

Also, another respondent (Ogoni chief [OC1]), commented that:

‘women are not very involved in the ongoing clean-up. If you check the records on site it is only one woman probably a nurse that is a woman on site’. This is corroborated during the FGD by OC4 who also noted that “on almost all the sites being remediated, the highest number of women you will see working on the lots is two; a cleaner and a nurse”.

This has also generated dissatisfaction amongst women in the study communities as captured by the position given by OC7 who noted that:

“ever since this clean-up started, women have not been given any consideration. We are the ones that can no longer go farm, we are the ones that can no longer take care of our families, we are the same people that can no longer cultivate cassava on our farmland, yet HYPREP cannot see it to be important to come to our community to ask us what we want them to do for us”.

The acknowledgment of the administrative and systematic exclusion of women in the clean-up process is also observed by men as Ogoni male1 (OM1) noted that:

“women involvement opportunity was taken over by men, for instance during the Livelihood campaign carried out by HYPREP, men took over the town hall and were the ones nominating people and writing names without giving women opportunity to contribute or lead the process themselves so even from the community level it is all men affaires. Women were taken over by men the Chiefs; they take over even the selection process that should be done by women”.

These outcomes and positions when coalesced, have had implications for opportunities for and actual participation of women in the clean-up process.

### Consultation of Women in the Operations of HYPREP in Communities

**Fig. 2:**
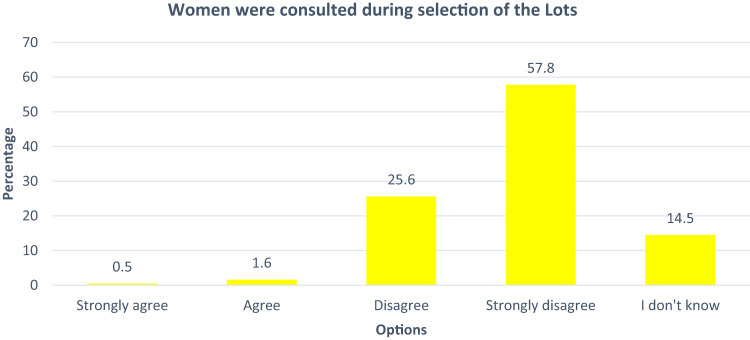
Consultation of women in the operation of HYPREP in communities

The distribution of responses to the statement that women were consulted during the selection of lots to clean-up is instructive. Only 0.5% of the respondents strongly agreed that women were consulted; respondents who agreed with the assertion on the consultation of women are 1.6% (Fig. 2). 25.6% on the other hand disagreed and 57.8% strongly disagreed while 14.5% did not know whether women were consulted (Fig. 2).

### HYPREP Provision of Avenues for Women Engagement in the Clean-up Process

**Fig. 3:**
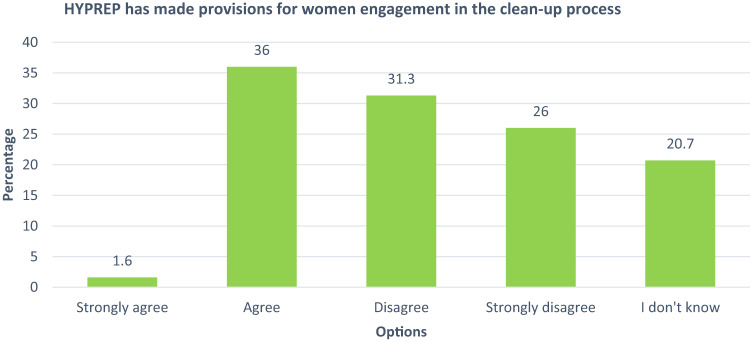
Perception on the provision made by HYPREP for women engagement in the remediation process

As captured in Fig. 3, 36% of the respondents agree that HYPREP made provisions for women engagement in the clean-up process; 31.3% disagree with the statement and 26% strongly disagree. The results (Fig. 3) revealed that over 50% of the respondents believe that HYPREP has not made provisions for engaging women. 20.7% didn’t know if HYPREP made provisions for women participation in the clean-up process

### The Role of Women in the Remediation Process

Given the cultural context of the role of women as caretakers and household managers in Ogoni, this research explored potential roles women could play in the ongoing Ogoni remediation exercise.

**Fig. 4:**
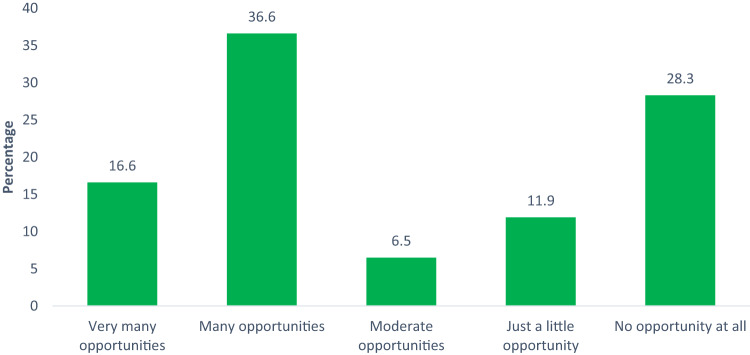
Women perception on the availability of opportunities for participation

The respondents identified that opportunities for women participation in the clean-up process exist. 16.6% note that very many opportunities exist; 36.6% hold that many opportunities exist (Fig. 4). 6.5% claim that moderate opportunities exist while 11.9% note that just a little opportunity exist and 28.3% claim there are no opportunities at all (Fig. 4). As the findings show, majority of respondents (>70%) agreed that opportunities for women to play a role exist (Fig. 4). This position was corroborated by OF2 during the FGDs.

“Yes, there are opportunities, but HYPREP is not open to women”. Others also maintained that “there are opportunities within HYPREP (men and women can be trained as monitors” and “Women leaders in Ogoniland can mobilize community women in Ogoni for HYPREP to engage”.

### Opportunities for Women Participation in the Clean-up Process

Considering that women are disproportionately impacted by oil spills in the context of Ogoniland, there should be opportunities for them to participate in the remediation of polluted sites in their area. Women are willing to work at the sites, provide security, sensitize the community on the remediation project, undertake administrative roles in HYPREP, and provide support roles to site workers (Table 4).

On available opportunities for women participation in the clean-up process, respondents identified several opportunities including women working at the clean-up lots, 1.6%; provide security at sites, 19.7%; agents of sensitization on local knowledge and processes, 3.3%; function as liaison officers, 1.6%; provide catering services, 3.3%; provide support roles for other clean-up site workers, 4.9%; and provide administrative roles 1.6%. As women themselves noted, there are several opportunities for them to support the clean-up process. For example, OM6 commented thus:

“Women can play a whole lot of roles technical role, labor supply, site staff, security work, nurses etc.”. Another respondent and FGD participant noted that “whatever role men play women can equally do so. Women are doing Peace building work. They can address insecurity through peacebuilding campaigns”. Another participant noted that “Women could be involved in decision making process /governance do administrative jobs, provide labor and technical services, women can be mediators and do peace building work”.

### Stakeholders’ Recommendations for Inclusive Environmental Decision-making

The study interrogated potential avenues for enhancing inclusive environmental decision making as part of the Ogoni clean-up program. In this regard, the study queried avenues for strengthening the relationship between HYPREP and representative bodies of women groups. The responses are captured in Table 4.

In terms of strengthening the working relationship between women groups and HYREP, recommendations given by the respondents spanned across several participatory themes (Table 4). One such avenues identified is the funding of network capacity building training, 7.1%; advocacy visits for enhancing working relationships, 21.4%; women’s group meetings as interest articulation channels 7.1%; Community town hall meetings as interest aggregation channels, 7.1%; petitioning the government, 2.9%. However, even with these highlighted avenues, the issue of apathy amongst women in participating in decision-making is still high as 22.9% prefer to do nothing and take no action in enhancing inclusive environmental decision making in the context of the clean-up exercise.

## Discussion

### Current Level of Women Participation in Decision-making in the Cleanup Process

The data from the survey and the FGDs show low levels of women participation in the clean-up process (Table 2). This could be attributed to different factors including but not limited to level of awareness of the clean-up exercise, lack of trust in government policy, and lack of community ownership of the clean-up exercise (Sam et al. 2022). Notably, access to information is critical to civic engagement in the areas of decision-making and participation. This is important as it determines and shapes the extent of involvement and the nature of communication between stakeholders (Fung 2006). Sam et al. (2022) explained that limited sensitization, and awareness or the deliberate adoption of non-inclusive communication strategy have limited the effectiveness of women participation in the remediation project. Also, the UNEP recommendations being implemented was published in complex and scientific language requiring simplification for communities to understand (Sam et al. 2022). Between 2012 and 2016, HYPREP became defunct and was revitalized in 2017, creating in the process, a gap in communication and consultation with communities. This may have affected community trust in government to clean-up polluted areas in the Niger Delta region. Thus, community ownership of the project is yet to be achieved, which negatively affects women participation.

Practically, there are different avenues for women participation in the clean-up value-chain, particularly regarding economic empowerment. These opportunities include but are not limited to supply of remediation materials (e.g., laterite), consulting, contracting, local monitors, actual site clean-up and involvement in HYPREP’s governance structures (e.g., CRAC) (Sam et al. 2022; Ekpootu and Nbete, 2023). Considering the responsibility of CRAC and cultural role of women in the community (i.e., custodians of peace), they would have been most useful in CRAC.

In addition, majority of the respondents (93%) commented that women are not engaged as clean-up monitors and considered insignificant in the supply of materials used in the remediation process. Women therefore rated their participation very low across highlighted ventures as only a minority of respondents (Table 2). perceived women to be engaged in the broad spectrum of the remediation exercise depending on matching skill sets. These levels of exclusion are primarily attributable to limited awareness of, and lack of clear gender policy which affects project ownership by women and their communities. Again, it shows a systemic arrangement where women are adjudged to be suitable for limited and predetermined roles outside core decision making structures regarding the clean-up exercise.

These issues account for the dissatisfaction of majority of women in the remediation process, as corroborated by Bodo and Ukpong (2018). When asked during the FGD to identify reasons for the dissatisfaction; the reasons identified included perceptions around incidental economic disadvantages arising from impacted natural capital, social discrimination, and expectations of the socio-economic benefits of the remediation project that has not been met. The data outcome indicates a mismatch of expectation between women and HYPREP’s socio-economic deliverable of the remediation project to the communities (Sam et al. 2022). Limited understanding, interaction and integration of women’s expectations and needs could and have systematically resulted in exclusion and dissatisfaction (Bodo and Ukpong 2018; Giadom and Wills 2021).

Given the political context of the clean-up, transparency and clarity of purpose could also affect participation. Discussions during the FGDs show that community folks are dissatisfied with the convoluted administrative processes and the non-transparent recruitment processes (Bodo and Ukpong 2018). With such general perceptions about the project and how it is administered, the implication is that the process is neither participatory nor transparent (Sam et al. 2022). Similarly, HYPREP seems to have adapted perennial cultural practices that bars women from active involvement in decision-making processes in Ogoni communities, and thus limit the robustness of most decisions reached (Sam and Zabbey 2018). This means that the formal administration of the project has been designed to embed and sustain exclusionary and non-participatory models. Women are excessively dominated, intimidated and coerced which often results in withdrawal, even at community level meetings.

The implication is that at different levels of opportunities for participation and decision-making in the clean-up process, women are disadvantaged and do not have the necessary support to join in, participate, and contribute to the process. The seeming exclusion could also be attributed to the failure to acknowledge the capacity of women to contribute meaningfully to remediation decision-making process (Ekpootu and Nbete, 2023, given that it is a technical exercise.

Beyond the involvement of women in the clean-up process by HYPREP, women commented on exclusion by the community leadership from the community governance structure (Kpae 2021). This highlights the patriarchal context within which the project exists. It precludes considerations of equity in decision-making, considering the traditional economic practices of farming being dominated by women (Sam et al. 2022), and could be a source of uncertainty in the decision-making process (Sam 2023). This sense of suppression and intimidation is a potential setback for women participation in the decision-making. It is therefore imperative for community leadership to ensure women involvement in decision-making processes, so as to build their confidence and capacity in participatory multi-stakeholder initiatives such as the Ogoni clean-up project.

### Consultation of Women in the Operation of HYPREP in Communities

Another significant area of concern is the issue of consultation. Consultation is a precursor to participation (Awung and Marchant 2018; Kahangirwe 2011; Johnston 2010). When women consultation by HYPREP was assessed (Fig. 2), the data shows that women in impacted communities are not adequately consulted during the contaminated site selection and prioritization for remediation (Sam et al. 2017a).

The distribution of responses (Fig. 2) on the consultation of women is significant in a number of ways (Fig. 2). Firstly, the Ogonis are traditionally a fishing and farming population (Pegg and Zabbey 2013). Collective and productive farming is highly dependent on the availability of arable land. More importantly, women folk engage more in farming even with the prevailing soil sterility caused by oil pollution (Sam et al. 2017a). This implies that women understand the intricacies regarding impacts of pollution and could contribute indigenous knowledge to the risk assessment and characterization of such sites (Sam 2023).

Secondly, the selection process should be representative, including all segments of the Ogoni society as land is a collective resource. Determining the sites to be prioritized for remediation should be inclusive i.e., involve every aspect of the communities affected (Sam et al. 2017a). This has significant implications for the success of the project as it has been noted that the nature and extent of consultations in environmental impact projects have far reaching implications for the success or failure of such projects (Kahangirwe 2011). Drawing from the nature of the remediation project, consultation and actual engagement are systematically segmented. Importantly, the data shows a lack of inclusive engagement in the consultation phase of the project.

### HYPREP Provision of Avenues for Women Engagement in the Clean-up Process

The data on Fig. 3 implies that while the provisions for women engagement may exist, knowledge of its existence is limited amongst women in the communities. This outcome is understandable when juxtaposed with initial data outcomes that showed limited access to information on the remediation project (see Fig. 2). According to Sam et al. 2017, the use of appropriate communication channel and mechanisms is critical to relaying and engaging stakeholders in environmental decision-making. However, FGD data shows that the pathways for HYPREP to engage women is unclear and burdened by bureaucratic issues. Women engagement in the remediation process would benefit from deliberate communication strategy that suits stakeholder needs, which is currently lacking in the Ogoni remediation project (Zeeuw et al. 2018).

### The Role of Women in the Remediation Process

Culturally women are placed to be caretakers and household managers. To achieve household responsibilities, they are mainly involved in farming and fishing to feed their families and access finance for sustenance. This role is increasingly difficult considering that soils are no longer fertile to support farming and the rivers not able to support fish breeding due to pollution (Sam et al. 2017). This cultural role has been reported as a rationale for the unconscious exclusion thereby affecting their opportunities to contribute or express their opinions or make decisions towards the remediation exercise (Catalán-Vázquez et al. 2012).

The data indicates that women are optimistic to participate by playing different roles to contribute to successful remediation project. While the data shows a majority of the women perceive that many opportunities for participation in the remediation process exist (Fig. 4), the peculiarity of the environment emphasizes gender consideration during engagement which is further worsened by contextual security challenges. For example, women may be perceived not to be physically built for confrontation if a conflict situation occur on site, as such, it is safest to keep women away from the remediation area. This sort of decision could also be based on culture -where strenuous jobs, such as remediation exercise, are reserved for men. However, for a tensed environment like Ogoniland with latent security issues, women play critical roles in supporting and building peace, sensitization and awareness creation.

A critical issue to consider when scoping available opportunities for women participation in the clean-up process is the specific skills and capacities required to add value to the process. These skills may not be evenly distributed across gender considerations at the time of engagement. Nonetheless, it indicates far deeper perceptions around the discrimination arising from gender considerations of specific professional skillsets and occupations. Essentially, such notions often are no representation of actual gendered professional skill distribution in Ogoni.

### Opportunities for Women Participation in the Clean-up Process

We compared the current level of women participation (Table 2) with available opportunities for participation (Table 3) considering the population of women in specific remediation sites and communities. This provided a strategic framework for assessing interventions and women participation so that no group or section is disadvantaged. Where the current level of participation does not tally with the available opportunities for participation, the interventions should focus on linking women with more opportunities, supported by the active participation of HYPREP and community leadership.

**Table 3:** Available Opportunities for Women Participation in the Clean-up Process

		Frequency	Percent	Valid Percent	Cumulative Percent
Valid	work at the Lots	1	0.3	1.6	1.6
	Provide security at the site	12	3.1	19.7	21.3
	Sensitize HYPREP on local knowledge and process	2	.5	3.3	24.6
	Monitor activities at the Lots	1	.3	1.6	26.2
	Function as Liaison Officers	1	.3	1.6	27.9
	Provide housekeeping services	2	.5	3.3	31.1
	Provide support roles for site workers	3	.8	4.9	36.1
	Admin roles in HYPREP	1	.3	1.6	37.7
	Others	1	.3	1.6	39.3
	Total	61	15.6	100.0	

The intent is that such an approach will instrument the commitment and willingness of women to participate, not merely for economic reasons, but for informed decision-making by the authorities managing the remediation exercise. Building on this research, further work needs to explore socio-cultural and environmental barriers hindering women’s participation in the remediation exercise.

### Stakeholders’ Recommendations for Inclusive Environmental Decision-making

Opportunities for strengthening and bolstering women’s participation in the remediation process (Table 4), were outlined by respondents during data collection engagements. These include specific advocacy visits, building enhanced networks, capacity building and holding townhall meetings with women and disadvantaged groups (Table 4). Evidence suggests that women groups are aggrieved, frustrated and thus not interested in environmental decisions. This could be attributed to many failed promises of environmental remediation and restoration in the area (UNEP 2011). However, focused advocacy visits are most preferred for strengthening engagement and relationship between women and environmental decision-makers. Intentional advocacy visits should be used to relay clear information about environmental projects to excluded groups and opportunities for participation in decision-making outlined. Feedback from such engagement should be integrated in final decision to build trust, confidence and project ownership (Sam et al. 2017b). Also, creation of new networks could present opportunities for communicating and contacting excluded groups to participate in environmental management engagements. For example, the Centre for Environment, Human Rights and Development, a community-based organization created the women in environmental justice network (CEHRD 2023). The network is a platform created to disseminate information to women and derive feedback for decision-making. Deliberations and decisions made in the group is insulated from cultural practices such as male dominance. Creating such platforms would improve confidence, develop capacity and empower women for inclusive environmental decision-making.

**Table 4:** What can be done to Strengthen the Relationship between Women Groups and HYPREP?

		Frequency	Percent
Options	Forming a network	11	15.7
	Capacity building training	5	7.1
	Advocacy visit	15	21.4
	Discussed the issue(s) at a women’s group meeting(s)	5	7.1
	Voiced out at community town hall meeting	5	7.1
	Sign petition to government	2	2.9
	Join community protest / demonstration	1	1.4
	Media briefing	1	1.4
	Hold Ogoni Women Forum and invite HYPREP	2	2.9
	Do nothing	16	22.9
	Other	4	5.7
	Total	70	100.0

Creation of network could serve many purposes. Firstly, because women are already in groups (Ekpootu and Nbete, 2023), mobilization for environmental decision-making becomes easier. Group communication could enhance speedy transfer of information and encourage debates and exchanges that facilitate information. Secondly, working in groups create opportunity for knowledge sharing, capacity building and co-creation of sustainable solutions to environmental decisions (Sam et al. 2017c). As group members debate topical issues, they develop confidence and capacity to discuss, interrogate and explore benefits of environmental decision-making. However, achieving this is dependent on trust between stakeholders and confidence in the drivers of environmental decision-making process (Sam et al. 2022). Considering the benefits of inclusive and participatory decision-making in the Ogoni remediation process, HYPREP and CSOs need to deliberately develop programs to regain stakeholder trust and confidence in the process.

There is also need to strengthen social cohesion between stakeholders. Mobilizing women to participate in environmental decision-making process could be challenging where social cohesion is weak (Malek et al. 2021). This could be enhanced when environmental stakeholders take deliberate interest in issues that concern women (Sam et al. 2017c). For example, participating in women festivities particularly when they are invited. During the FGDs participants maintained that HYPREP has declined several invitations to engagements and activities executed by women groups, including rejections to participate in a tree planting exercise during the 2020 World Environment Day. Women construed this as unwillingness of key duty bearers to enhance social cohesion.

Developing a gender policy that outlines a clear strategy to women inclusion in the remediation process could help HYPREP to achieve greater environmental outcomes (Sam et al. 2022). This could imply specifying percentage of women inclusion in remediation process and taking necessary steps towards achieving it. This could also involve initiating women capacity development programs, and creation of networks or communication platforms for women. Of course, this will generate interest in women and motivate them to participate in the remediation process. Also, adapting a suite of communication strategies for reaching several women groups and platforms could enhance information sharing and bolster confidence in environmental issues.

## Conclusion

In this research we have demonstrated that women have roles and can participate in environmental decision-making processes particularly in the remediation of sites polluted by petroleum hydrocarbon in developing countries like Nigeria. What this shows is that inclusivity in decision making needs to be understood from two perspectives. Categorization and degree of participation. The identification of participants and the available opportunities for them to participate should include a broad-spectrum of the communities, more than just a selective few in environmental decision-making. We must also consider the degree of participation. Decision-making should be holistic and should cover all aspects of the remediation process. Any disconnection in decision-making in the different phases of the clean-up process may have long-term negative impacts on specific sections of the society as community folks attempt to reposition themselves in the emergent everyday livelihood practicalities arising from remediation.

## Recommendation

Currently, women participation in environmental decision-making is low even as identifiable roles and opportunities for inclusion exist. These have been shaped by extant sociocultural processes around community governance. From an institutional perspective, the administrative architecture of HYPREP replicates cultural practices that restrict women which primarily functions to limit inclusivity in environmental decision-making. As indicated in the analysis, opportunities exist for women involvement in the administrative structures, peace building process, sensitization and awareness creation, and the technical remediation process. Women can consult, supply materials and provide support to workers on remediation sites. This is however currently barred by the lack of skills, weak communication and engagement strategies, lack of gender policy and the limited commitment to mainstream women in the remediation process in Ogoniland. Different stakeholders including HYPREP, CSOs, development partners, community-based organizations and government have different roles to play to improve women participation and inclusion in the environmental decision-making processes. We recommend addressing the issue of inclusive decision-making from a multi-level policy perspective starting with HYPREP, and then the government at all levels.
